# Global identification of genes and pathways regulated by Akt during activation of T helper cells

**DOI:** 10.12688/f1000research.2-109.v2

**Published:** 2013-05-15

**Authors:** Jing Cheng, Lawrence P Kane

**Affiliations:** 1 Department of Immunology, University of Pittsburgh School of Medicine, Pittsburgh, PA, 15261, USA

**Keywords:** T helper cell; global microarray analysis; allosteric inhibitor; TFactS; CD28; CD3; Akt; NF-kB

## Abstract

We previously demonstrated that Akt differentially modulated a subset of NF-kB target genes during T cell activation. In the current study, we further explored the broader effects of Akt inhibition on T cell gene induction. Global microarray analysis was used to characterize T helper cell transcriptional responses following antigen receptor stimulation in the absence or presence of Akti1/2 (an allosteric inhibitor which targets Akt1 and Akt2), to identify novel targets dependent upon Akt and obtain a more comprehensive view of Akt-sensitive genes in Th2 helper T cells. Pathway analysis of microarray data from a CD4 ^+^ Th2 T cell line revealed effects on gene networks involving ribosomal and T cell receptor signaling pathways associated with Akti1/2 treatment. Using real-time PCR analysis, we validated the differential regulation of several genes in these pathways, including *Ier3*, *Il13, Egr1*, *Ccl1* and *Ccl4*, among others. Additionally, transcription factor target gene (TFactS) analysis revealed that NF-kB and Myc were the most significantly enriched transcription factors among Akt-dependent genes after T cell receptor and CD28 stimulation. Akt activation elicited increases in the enrichment of NF-kB- and Myc-targeted genes. The present study has identified a diverse set of genes, and possible mechanisms for their regulation, that are dependent on Akt during T cell activation.

## Introduction

Akt/protein kinase B (PKB) is a serine/threonine kinase that is activated downstream of PI-3 kinase. The activation of Akt leads to the phosphorylation and regulation of a wide spectrum of substrates involved in multiple cellular processes, including cell survival, growth, differentiation, cell cycle progression, proliferation and metabolism ^1^. The expression of a constitutively active form of Akt (myr-Akt) in transgenic mice was reported to influence thymocyte selection, lead to accumulation of CD4 ^+^ T cells in peripheral lymphoid organs, and to enhance T cell survival in the presence of various apoptosis-inducing stimuli ^2^. We have previously studied the role of the Akt kinase in T cells, and most recently showed that a subset of NF-κB-dependent genes required Akt for optimal upregulation during T cell activation ^3^. Studies of individual transcription factors and their target genes have uncovered numerous aspects of Akt signaling in T cells, including regulation of not only NF-κB, but also FOXO and NFAT ^4– 9^. However, the overall gene expression program controlled by Akt signaling in activated helper T cells has not been elucidated. Mapping global changes in gene expression has proven extremely useful in revealing previously unappreciated connections between groups of expressed genes and biological events, such as development and tumorigenesis. A study from the Cantrell lab ^10^ concluded that the chief role of Akt in CD8 ^+^ cytotoxic T cells is to control the transcriptional programs that direct effector versus memory cell fate. Nonetheless, Akt may not have the same role in all T cell subpopulations. For example, constitutively active Akt can stimulate the growth and survival of CD4 ^+^ T cells but not CD8 ^+^ T cells ^11, 12^.

In the current study, we tested the effects of a selective, allosteric inhibitor of Akt1 and Akt2 (Akti1/2) ^13– 16^ on activated T cells and further explored potential mechanism of action of Akt, by performing network analysis of gene expression data and validating the expression changes of selected genes by real-time qPCR analysis. Our findings demonstrate that Akt inhibition by Akti1/2 significantly affects ribosomal protein expression and the cytokine-cytokine receptor interaction gene expression axis. Asthma and the antigen receptor signaling pathways were also impaired by Akti1/2 in activated T cells. Moreover, Akt inhibition decreased the enrichment of NF-κB- and Myc-targeted genes after CD3/CD28 stimulation. These effects may contribute to the functions of dysregulated Akt activation in tumorigenesis, as well as in normal T cell activation and development ^12^.

The importance of Akt for T cell activation and transformation led us to explore the underlying pathways and mechanisms (or target genes and downstream cellular pathways) by a genome-wide gene expression profiling approach. Therefore the aims of the present study were to (1) identify, at the genome-wide level, the genes that are differentially expressed in activated Th2 helper T cells, with or without Akt inhibition and (2) conduct bioinformatics analyses to identify the pathways and possible mechanisms involved.

## Materials and methods

### Antibodies and reagents

Biotin-anti-mCD28 (37.51) and biotin-anti-mCD3ε (145-2C11) were obtained from BD-Biosciences (San Jose, CA). Streptavidin was obtained from Invitrogen (Carlsbad, CA) and Akti1/2 was from EMD Biosciences (San Diego, CA). rhIL-2 was obtained through the NIH AIDS Research and Reference Reagent program, Division of AIDS, NIAID, NIH (Cat.#136 from Hoffman-LaRoche, Inc.).

### T cells

The D10 T cell line, a fast-growing variant of the D10.G41 murine Th2 T cell clone ^17– 19^ was maintained in RPMI 1640 media (Mediatech, Manassas, VA), supplemented with 10% heat-inactivated bovine growth serum (BGS; Hyclone, Logan, UT), 0.1 mM nonessential amino acids (Lonza, Walkersville, MD), 2 mM l-glutamine, 50 µM 2-ME, 100 U/ml penicillin, 100 µg/ml streptomycin (Mediatech, Manassas, VA) and 25 IU/ml rhIL-2.

### RNA extraction and microarray gene expression profiling

D10 T cells were left untreated or pretreated with 10 µM Akti1/2 for 1 h and then stimulated with biotinylated anti-mCD3/CD28 and streptavidin for 0, 2, 6 and 12 hrs. RNA extraction was performed using a commercially available kit (RNeasy, Qiagen, Frederick, MD) according to the manufacturers’ recommendations. RNA quality was confirmed based on a RNA integrity number >8 by use of the Agilent 2100 bioanalyzer (Agilent Technologies, Palo Alto, CA). The microarray analysis was performed by Genomics and Proteomics Core Laboratories (GPCL) of the University of Pittsburgh, USA. An Illumina mouse RefSeq8 chip was used. Microarray data have been deposited in the GEO database and are accessible through the GEO series accession number GSE45221.

### Statistical analysis of gene expression microarray data

To compare the molecular characteristics between different time points, Automated Efficiency Analysis was first performed using 7 transformation methods, 9 normalization methods and 5 tests for differentially expressed genes ^20^. A global normalization method and the J5_Quantile95_None method were applied on each time point. The differentially expressed genes were identified using caGEDA with a reasonable threshold of J5 for each time point ^21^. To survey the spectrum of biological functions within genes, which were differentially expressed between different groups, functional classification of the genes were performed using Pathway Express ( http://vortex.cs.wayne.edu; a pathway level Impact Analysis as described by Draghici *et al.*, 2007 ^22^). Pathway Express was designed to provide both statistical and biological significance in the indication of which pathways may be affected by the observed changes in gene expression. The results are summarized as Impact scores and p-values. Pathway-Express orders the affected pathways in the decreasing order of their expected importance for the given condition.

### Real-time PCR analysis

Quantitative real-time PCR was performed using the ABI Step One Plus Real-time PCR system (Applied Biosystems, Foster City, CA) as described previously ^3^. Amplification was performed on a cDNA amount equivalent to 25 ng total RNA with 1×SYBR green universal PCR Master mix (Applied Biosystems) containing deoxyribonucleotide triphosphates, MgCl _2_, AmpliTaq Gold DNA polymerase, and forward and reverse primers. Specific primers for each gene were purchased from SABiosciences (Qiagen, Frederick, MD). Experimental samples and no-template controls were all run in duplicate. The PCR cycling parameters were: 95°C for 10 min, and 40 cycles of 94°C for 15s, 60°C for 1 min. The amount of cDNA in each sample was calculated by the comparative threshold (Ct) method and expressed as 2exp (Ct) using 18S RNA as an internal control. Statistical significance was determined using the Student’s T test. All statistical tests were performed using GraphPad Prism (GraphPad Prism, San Diego, CA).

### Enrichment in transcription factor target gene analysis

TFactS was used to predict the activities of transcription factors in our microarray data ^23^. The lists of up- and down-regulated genes were compared to a list of curated target gene signatures. The nominal p-value (Pval) represents the risk of a false positive for a single test. Since the list of query genes is systematically compared to each target gene signature, a multi-testing condition is required. The e-value (Eval) represents the expected number of false positives for a given nominal value. It is computed using the formula: Eval=Pval*T, where T is the number of tests.

## Results

### Identification of genes regulated by Akt signaling in activated CD4 ^+^ T cells

**Figure 1.**
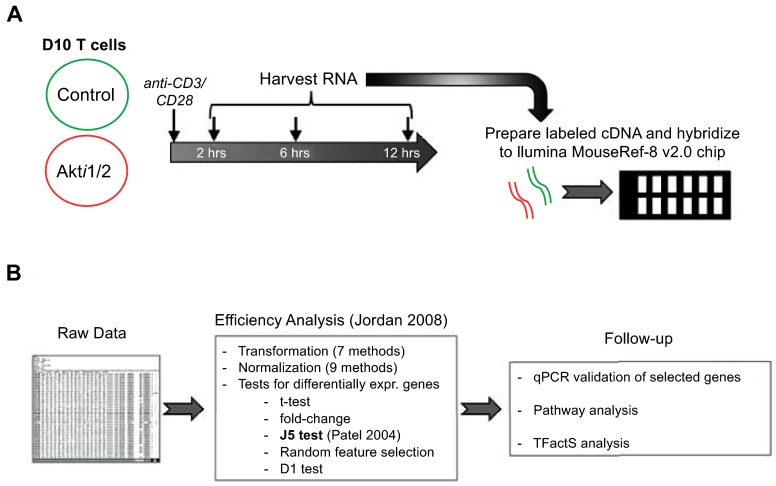
Flowchart of experimental outline ( **A**) and data analysis ( **B**), as described further in the text.

We previously demonstrated that Akt activity was rapidly inhibited in T cells by addition of the allosteric inhibitor Akti1/2, which inhibited phosphorylation of Akt within one minute, an effect that can last as long as twelve hours ^3^. In the present study, microarray analysis was performed at three different time points after CD3/CD28 stimulation to characterize the effects of Akt inhibition on the T cell gene activation program in helper T cells. Thus, D10 T cells (a murine Th2 T cell line) were pre-incubated with 10 μM Akti1/2 or solvent, then stimulated for 2–12 hours. We chose this concentration of inhibitor for two reasons. First, in our recent study we observed good concordance between results obtained with 10 μM Akti1/2 and those obtained with combined siRNA-mediated knock-down of Akt1 and Akt2 ^3^. In addition, although 1–5 μM can substantially inhibit Akt activity in different cell types under acute conditions ^24, 25^, at least one study has demonstrated that a higher concentration (10 μM) of Akti1/2 was required for more significant inhibition of Akt substrate phosphorylation over the course of several hours ^25^. This could be related to the fact that full-length Akt is only inhibited approximately 80% by 1 μM (and 90% by 10 μM) Akti1/2 in *in vitro* kinase assays, as shown in a kinase profiling study by Cohen and colleagues ^26^. After stimulation, mRNA was isolated, which was converted into labeled cDNA for hybridization to Illumina chips for microarray analysis ( Figure 1A). A rough analysis of the genes modulated in our study after six or twelve hours of CD3/CD28 stimulation (using the default settings with the GEO2R tool at the GEO database) revealed that of the top 30 genes in each case, seven were modulated to a nearly identical degree in the presence or absence of 10 μM Akti1/2. Thus, we were reasonably confident that Akti1/2, with this cell type, and at the concentration used in our study, did not have widespread, off-target, effects on gene transcription or cell viability.

**Supplementary Figure 1.**
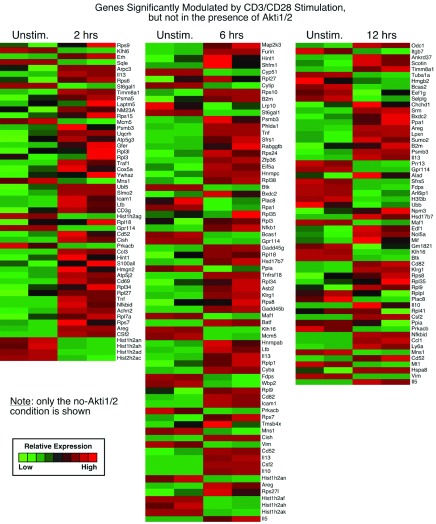
Genes significantly modulated by CD3/CD28 stimulation alone, but not modulated in the presence of Akti1/2. Each row represents a gene and each column shows one of the duplicates from two different groups (from left to right 0 h vs. 2 h, 0 h vs. 6 h, 0 h vs. 12 h). The red and green colors reflect high and low expression levels, respectively.

**Supplementary Figure 2.**
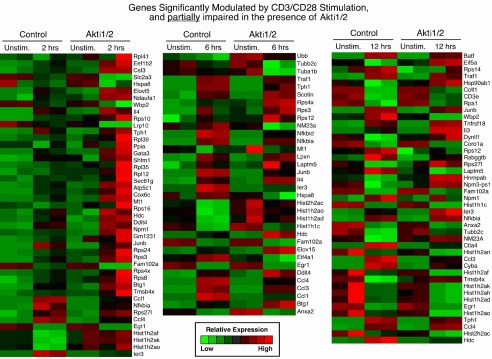
Genes significantly modulated by CD3/CD28 stimulation alone but less modulated in the presence of Akti1/2. Each row represents a gene and each column shows one of the duplicates from two different groups (from left to right 0 h vs. 2 h, 0 h vs. 6 h, 0 h vs. 12 h). The red and green colors reflect high and low expression levels, respectively.

**Supplementary Figure 3.**
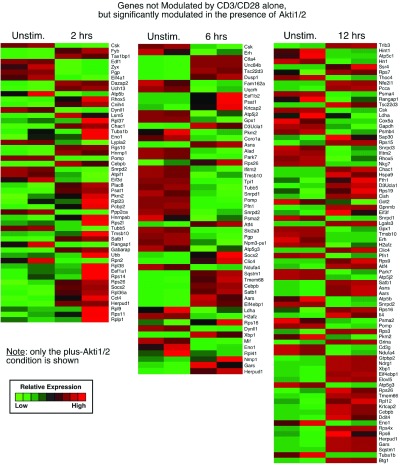
Genes significantly modulated by Akti1/2 alone. Each row represents a gene and each column shows one of the duplicates from two different groups (from left to right 0 h vs. 2 h, 0 h vs. 6 h, 0 h vs.12 h). The red and green colors reflect high and low expression levels, respectively.

We next determined which genes were differentially modulated after T cell receptor (TCR) stimulation using caGEDA with reasonably chosen thresholds for different time points (2 h, 6 h and 12 h). This methodology allows for the capture of a more complete set of differentially modulated genes, which is less dependent on overall expression levels. Further validation and downstream analysis were then performed to confirm some of the differentially expressed genes and to extract functional information from the dataset ( Figure 1B). We identified differentially expressed gene sets that were dependent on Akt among the three different time point groups. We compared the gene expression patterns of cells plus or minus addition of Akti1/2. First, we generated two gene lists for each time point. Gene list one represents the genes that were differentially expressed between the unstimulated and CD3/CD28 stimulated group in the absence of Akti1/2. Gene list two represents the genes that were differentially expressed between the unstimulated and CD3/CD28 group in the presence of Akti1/2. When comparing the two gene lists, three different patterns were observed:

1. Genes significantly modulated by CD3/CD28 alone but not modulated in the presence of Akti1/2 (genes in this category showed Akt–dependent expression after T cell activation; column 1, top, in Data File 1– Data File 3 and Supplementary Figure 1).

2. Genes significantly modulated by CD3/CD28 alone but less strikingly modulated in the presence of Akti1/2 (genes in this category showed some dependence on Akt; columns 1–2, middle, in Data File 1– Data File 3 and Supplementary Figure 2).

3. Genes not modulated by CD3/CD28 alone but significantly modulated in the presence of Akti1/2 (genes in this category displayed Akti1/2-specific expression; column 2, bottom, in Data File 1– Data File 3 and Supplementary Figure 3).

Genes with significant modulation at 2 hours of CD3/CD28 stimulation in the presence or absence of Akti1/2 Genes listed in the first sheet were significantly modulated (J5 score > 20 or < -20) at 2 hours after CD3/CD28 stimulation, but not significantly modulated (J5 score < 20 or > -20) after stimulation in the presence of Akti1/2. Genes listed in the middle sheet were significantly modulated by 2 hours of CD3/CD28 stimulation in the presence or absence of Akti1/2, but were nonetheless affected by the inhibitor. Genes listed on the last sheet were significantly modulated by 2 hours of CD3/CD28 stimulation only in the presence of Akti1/2.

Genes with significant modulation at 6 hours of CD3/CD28 stimulation in the presence or absence of Akti1/2 Genes listed in the first sheet were significantly modulated at 6 hours after CD3/CD28 stimulation, but not significantly modulated after stimulation in the presence of Akti1/2. Genes listed in the middle sheet were significantly modulated by 6 hours of CD3/CD28 stimulation in the presence or absence of Akti1/2, but were nonetheless affected by the inhibitor. Genes listed in the last sheet were significantly modulated by 6 hours of CD3/CD28 stimulation only in the presence of Akti1/2.

Genes with significant modulation at 12 hours of CD3/CD28 stimulation in the presence or absence of Akti1/2 Genes listed in the first sheet were significantly modulated at 12 hours after CD3/CD28 stimulation, but not significantly modulated after stimulation in the presence of Akti1/2. Genes listed in the middle sheet were significantly modulated by 12 hours of CD3/CD28 stimulation in the presence or absence of Akti1/2, but were nonetheless affected by the inhibitor. Genes listed in the last sheet were significantly modulated by 12 hours of CD3/CD28 stimulation only in the presence of Akti1/2.

By examining multiple time points after stimulation, we were able to obtain a kinetic picture of gene expression ± Akt inhibition. The 6 h Akti1/2 (+) and Akti1/2 (-) comparison showed the highest number of differentially expressed genes, and there were fewer differentially expressed genes after two or twelve hours of TCR/CD28 stimulation. Among these, only the genes that expressed the most consistent differences (either increased or decreased expression) were selected for further analysis. Genes with no known function were excluded.

**Figure 2.**
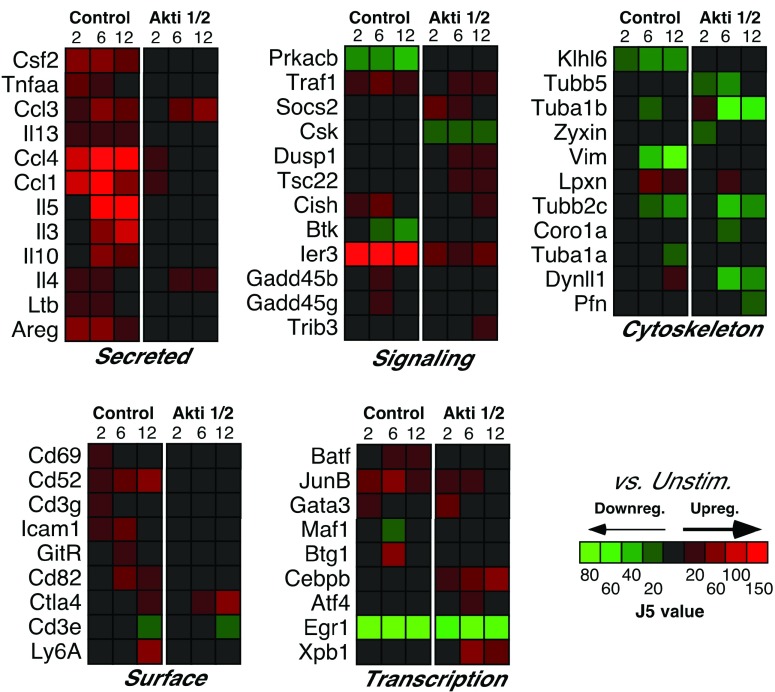
Selected Akt-dependent genes differentially expressed between control (0 h) and 2 h, 6 h and 12 h CD3/CD28 stimulation groups. Relative levels of expression are represented by the J5 score.

Our previous work identified several NF-κB target genes that were dependent on Akt after TCR stimulation in T helper cells, including those encoding the cytokines TNF-α, GM-CSF, and IL-10, among others ^3^. Analysis of the microarray data confirmed the dependency of these genes on Akt activation, which inspired confidence in our results. Moreover, expression of the mRNAs encoding many secreted proteins was also decreased by Akt inhibition, including IL-13, IL-5, IL-3 and IL-4 ( Figure 2). The protein products of these genes (except IL-3) were examined in our previous paper ^3^, which confirmed similar decreases after Akt inhibition. Our data agrees with Patra *et al’s* study ^7^, which showed that myr-Akt expression in activated CD4 ^+^ T cells resulted in increased Il-4 and Il-13 expression. In addition we found that expression of *Ltb* (encoding lymphotoxin β), *Areg* (encoding amphiregulin) and genes encoding the chemokines CCL1, CCL3 and CCL4 were also affected by Akt inhibition ( Figure 2).

Of note, several cell-surface proteins, including CD69, CD52 and CD82 were among the Akt-dependent genes after T cell activation ( Figure 2). The CD82 molecule is a type III integral membrane protein with four transmembrane domains and is part of the tetra-span-transmembrane (TST) family, which also includes CD9, CD37, CD53, CD63 and CD81/TAPA-1 ^27^. Interestingly, proteins of this family are involved in cell activation. For example, engagement of CD82 can deliver a co-stimulatory signal, similar to CD28, for full T cell activation, leading to strong IL-2 production ^28^. CD52 is a small glycopeptide molecule and tethered to the outer surface of the plasma membrane by a glycosylphosphatidylinositol (GPI)-anchor ^29^. CD52 crosslinking can also provide a co-stimulatory signal that causes the activation of normal human T lymphocytes ^30^ and induction of CD4 ^+^ regulatory cells ^31^. Since Akt was reported to mimic CD28 co-stimulation in a T cell line by synergizing with TCR-induced signals to increase transcription of the IL-2 promoter ^5^, activation of these other co-stimulatory molecules might also function through this pathway.

**Figure 3.**
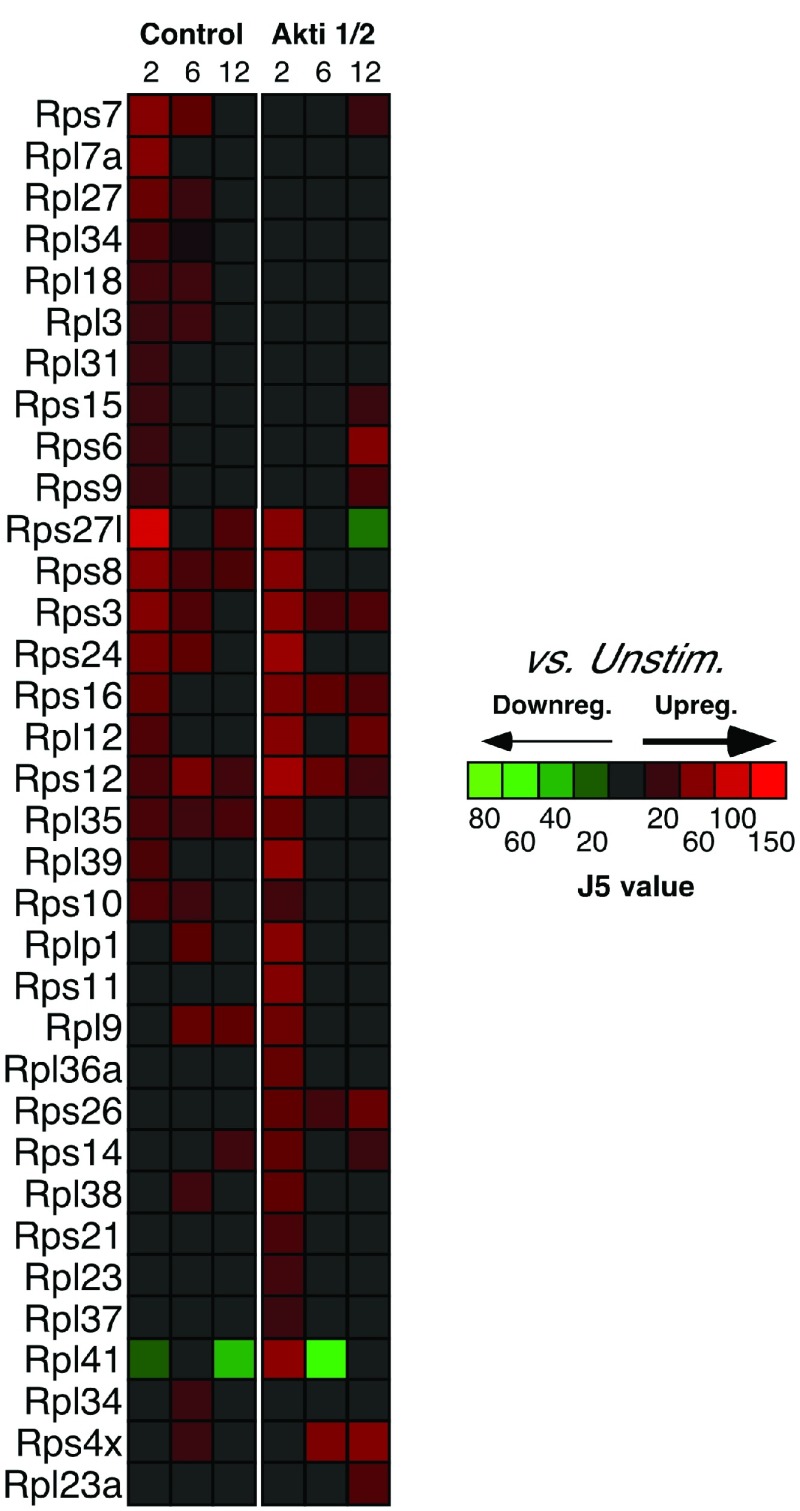
Akt-dependent ribosomal subunit genes differentially expressed between control (0 h) and 2 h, 6 h and 12 h CD3/CD28 stimulation groups. Relative levels of expression are represented by the J5 score.

Interestingly, inhibition of Akt resulted in impaired up-regulation of the genes encoding many ribosomal proteins ( Figure 3). These included: Rps6 (a major substrate of ribosomal protein kinases ^32^); Rps8, Rps9 (reported to be activated in various tumors, including colon cancer ^33^); Rps10, Rps15, Rps24 (mutations in these gene result in Diamond-Blackfan anemia ^34^); Rpl7a (which interacts with a subclass of nuclear receptors and inhibits their ability to activate transcription ^35^); Rplp1 (important for elongation during protein synthesis ^36^). All these proteins belong to either small or large subunits of ribosomes; changes in their expression may contribute to an increase in protein synthesis to accommodate numerous cellular processes involved in T cell activation, in addition to the possible connections to cancer. Importantly, the expression of 18S ribosomal genes, which was used for our qPCR housekeeping gene, did not differ between our experimental groups.

### Expression validation of selected genes

In order to validate the microarray data and obtain more quantitative data on specific genes, real-time PCR analysis was performed at different time points after CD3/CD28 stimulation ± Akti1/2. *Ier3* (also named IEX-1, immediate early response gene X-1), *Il13*, *Ccl1* and *Ccl4* were selected for real-time PCR validation because their expression was greatly enhanced after CD3/CD28 stimulation and decreased in the presence of the Akt inhibitor. Of note, the gene expression of *Ccl1* and *Il3* was rapidly up-regulated by CD3/CD28 stimulation, while the up-regulation of *Ier3* and *Ccl4* expression by CD3/CD28 stimulation was delayed. *Egr1* was also selected as a control gene, which showed no CD3/CD28 dependent increase in gene expression, but rather a sharp decrease. Thus, Akt inhibition could affect different genes that showed diverse kinetics after CD3/CD28 stimulation. At least for these specific genes, the changes in expression, as assessed by real-time PCR, confirmed the microarray results, giving us confidence in the overall quality of the dataset.

**Figure 4.**
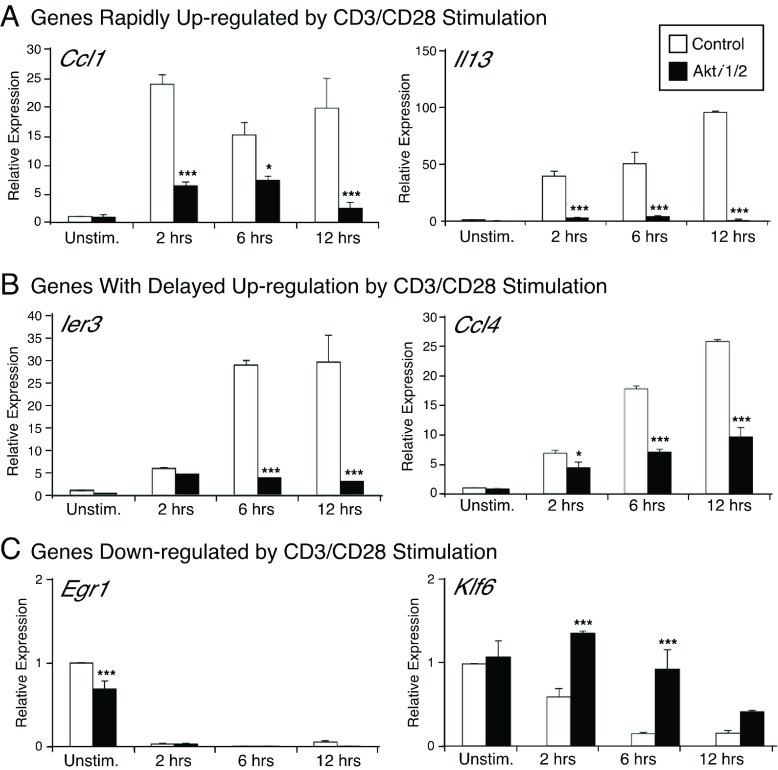
Quantitative real-time PCR (qPCR) confirmation of the stimulation-dependent expression of selected Akt-dependent genes. qPCR was used to validate the gene expression of *Ier3*, *Il13*, *Klf6*, *Egr1*, *Ccl1* and *Ccl4*. The RNA samples were the same as those used for the microarray. Results are presented as relative mRNA expression, compared to the unstimulated control sample, normalized to 18S RNA expression. ***p<0.001, *p<0.05 compared with the control group.

Akt is known to directly phosphorylate FOXO3a (including in T cells) and, once phosphorylated, FOXO3a is excluded from the nucleus and becomes transcriptionally inactive ^37^. One of the best characterized FOXO target genes in T cells is *Klf2* ^38^. For reasons that are unclear, we did not observe modulation of *Klf2* expression after CD3/CD28 stimulation, with or without Akti1/2. Among known or suspected FOXO target genes ^38, 39^, several that did display enhanced expression in our microarray experiment in the presence of Akti1/2 were *Ctla4, Gadd45, Cebpb* and *Klf6*. The latter was recently identified as a FOXO target gene, which was confirmed by chromatin immunoprecipitation ^40, 41^. Our own real time PCR analysis confirmed that in Akti1/2-treated samples, there was an up-regulation of *Klf6*, relative to stimulation with anti-CD3/CD28 alone, which by itself resulted in a sharp decline in the *Klf6* message ( Figure 4).

### Functional classification of the gene expression signature for Akt inhibition in activated T cells (pathway analysis of Akti1/2-mediated transcriptome changes)

From our previously published array data, we found that at two, six and twelve hours after CD3/CD28 stimulation. Akti1/2 elicited a wide range of effects on expression of numerous genes, including both over- and under-expressed genes. In the present study, genes that showed changes in expression after two, six and twelve hours of stimulation in the presence of Akti1/2 were largely not overlapping and cannot be combined for subsequent analysis. There were 54, 71 and 58 genes dependent on Akt at the two, six and twelve hour time points, respectively, after CD3/CD28 stimulation ( Data File 1– Data File 2 and Supplementary Figure 1).

**Figure 5.**
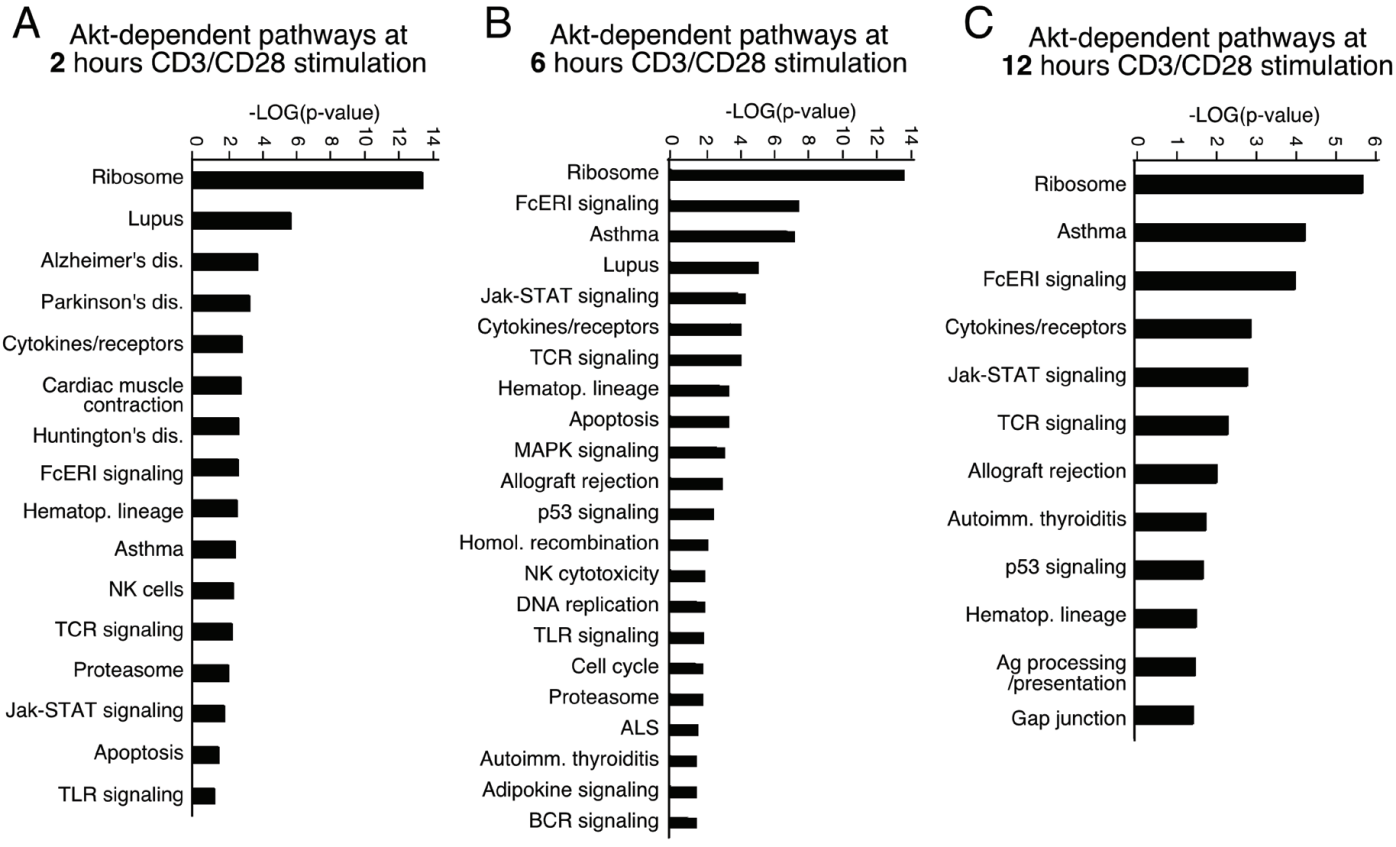
Functional classification of Akt dependent genes. Classification enrichment was determined using Pathway Express. Significance is indicated as –Log (p-value).

To determine whether our gene expression signature was enriched in specific subsets of genes with known biological functions, bioinformatic functional classification analysis of the genes that were differentially expressed was carried out as described in the Methods. The functional classifications of gene sets are illustrated in Figure 5. We found that genes involved in ribosome, cytokine-cytokine receptor interaction, antigen receptor signaling pathway, hematopoietic cell lineage and asthma were significantly enriched among genes affected by Akt inhibition in the presence of CD3/CD28 stimulation.

### Enrichment of transcription factors in target genes

**Figure 6.**
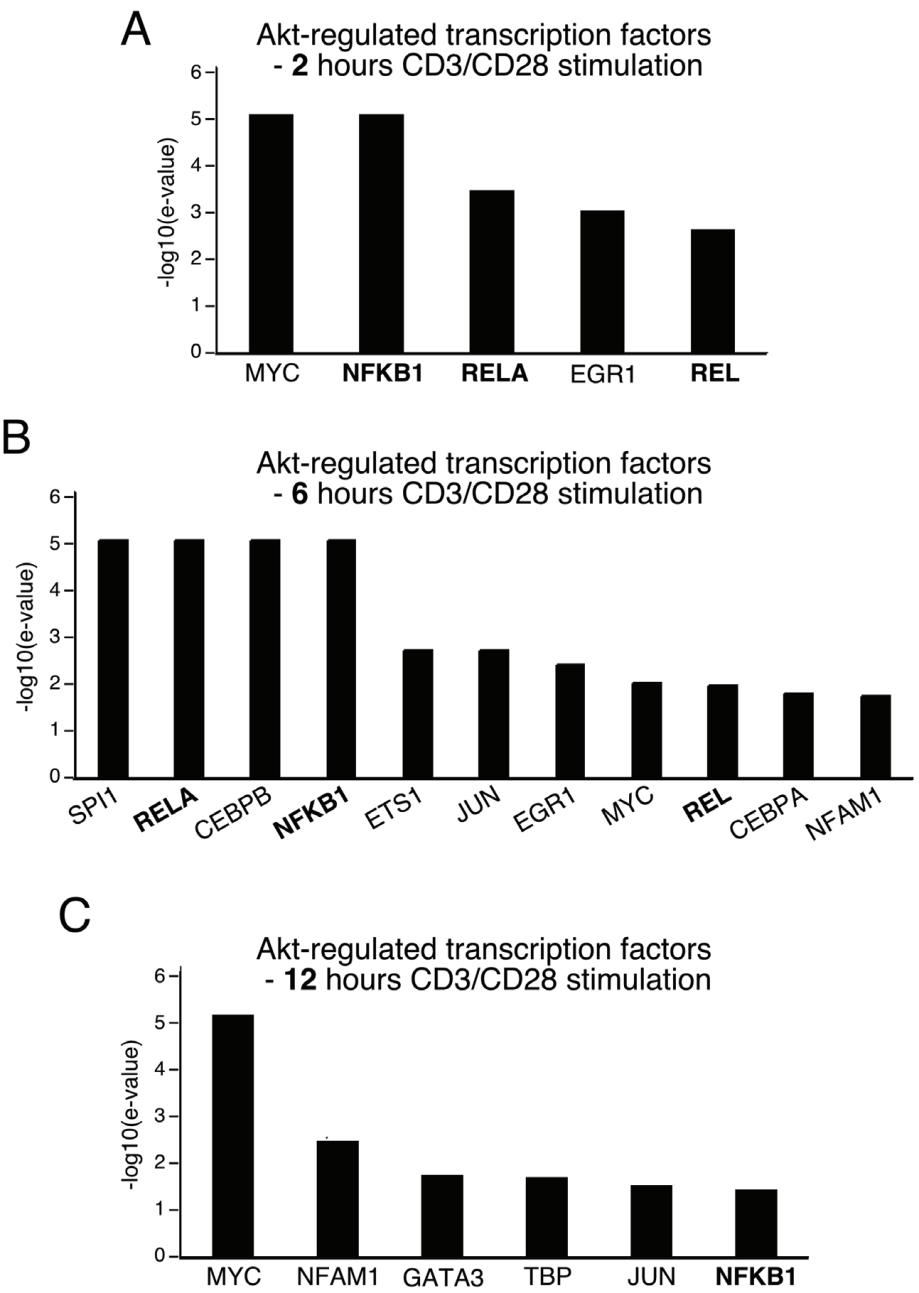
Transcription factor binding sites enriched in Akt-dependent genes, as predicted by TFactS. The list of Akt-dependent genes was submitted to TFactS (sign-less) using default settings. Significance of regulated transcription factors was determined with–log10 (e-value).

Co-expressed genes are often regulated by common transcription factors. Therefore, we separately analyzed the genes that showed altered expression at 2, 6 and 12 h time points after CD3/CD28 stimulation using TFactS, an algorithm that used a catalog of well-characterized transcription factor targets to predict the activity of transcription factors based on microarray data ^23^. Enrichment of transcription factors was found at all the time points tested ( Figure 6). These included NF-κB family members (RelA, Rel, NF-κB1), Myc, Jun and C/EBP. At the 2 h time point, Myc, NF-κB family members (Nfkb1, RelA and Rel) and Egr1 were significantly enriched. RelA, Nfkb1 and Rel, along with Myc and Egr1 were also significantly regulated after 6 h of CD3/CD28 stimulation. Moreover, other transcription factors, including Sp1, C/EBPB, Ets1, Jun and Nfam1, were also enriched at the 6 h time point. At 12 h after stimulation, however, fewer transcription factors were regulated, with Myc and NF-κB1 still significantly regulated. In summary, the most significantly enriched transcription factor binding sites in the regulatory regions of Akt-dependent genes after CD3 and CD28 stimulation were those of NF-κB family members and Myc.

## Discussion

Akt activation impacts the expression of genes responsible for cell proliferation and survival ^42, 43^. The majority of previous global gene expression studies have investigated transcriptional programs under the combined control of both PI3K and Akt ^44, 45^. Despite much attention in recent years to the role of Akt-regulated metabolism in T cells ^46^, a recent study provided clear evidence that Akt indeed contributes to changes in gene expression after activation of cytotoxic T cells ^10^. We have also been interested for some time in achieving a greater level of clarity in separating the effects of PI3K and Akt during helper T cell activation ^47^. Our recent study identified a subset of NF-κB-dependent genes that require Akt for optimal upregulation during helper T cell activation by using a targeted gene profiling approach ^3^. In the present study, we performed a broader microarray analysis to characterize the global changes in gene expression resulting from inhibition of Akt in activated CD4 ^+^ helper T cells. Analysis of the affected genes revealed pathways that are central to the effects of Akt on helper T cell activation. Finally, analysis of the enrichment in transcription factor binding sites in our target genes further confirmed NF-κB as a regulator of these genes in response to TCR stimulation.

The generation and maintenance of memory T cells is central to the development of protective immunity, as characterized by a rapid and vigorous response after a secondary encounter with a given pathogen or antigen ^48, 49^. Recent studies have suggested that proper regulation of Akt activity is essential for the development of memory T cells. Riou *et al.*, found that Akt plays a critical role in the phosphorylation of FOXO3 in CD4 ^+^ central memory T cells (T _CM_), thereby promoting T _CM_ survival ^50^. Abrogating the Akt survival pathway led to a greater degree of apoptosis in T _CM_ as compared with effector memory T cells (T _EM_), confirming that T _CM_ are more dependent on these pathways for their survival. Sustained and strong activation of Akt was also shown in CD8 ^+^ cytotoxic T cells (CTL) to coordinate the TCR and IL-2-induced transcriptional programs that control expression of key cytolytic effector molecules, adhesion molecules, and cytokine and chemokine receptors that distinguish effector from memory and naïve T cells ^10^. It has been suggested ^51^ that Akt simultaneously induces and represses expression of key genes, leading to the development of effector CTL, with the FOXO transcription factors being at the center of this process. Thus, Kim *et al.*, found that Akt appeared to function as a cellular rheostat, controlling distinct facets of the program that governed differentiation of Ag-activated CD8 ^+^ T cells into effector cells or memory CD8 ^+^ T cells ^51^. Myristoylated Akt transgenic mice were found to accumulate memory phenotype CD4 ^+^ T cells and to develop both tumors and autoimmunity ^52^, effects that could, in principle, be due in part to non-metabolic outcomes of Akt activation, such as NF-κB activation ^6^. In addition, FOXO transcription factors control the development and function of natural regulatory T cells (nTreg) ^53^, and the generation of inducible Treg (iTreg) is also regulated by both Akt and FOXO's ^54, 55^.

The enrichment of multiple NF-κB family members in Akt-dependent genes confirmed our previous study emphasizing the important role of NF-κB in Akt-dependent biological processes ^1^. Although FOXO did not appear in the list of the most significantly affected transcription factors in our analysis of Akt-dependent genes, expression of multiple FOXO target genes (including *Klf6*, *Gadd45* and *Ctla4*) was increased in the presence Akt inhibition, relative to stimulation with anti-CD3/CD28 alone. Using RT-PCR, we confirmed that Klf6 expression was decreased, as expected, by stimulation with anti-CD3/CD28 alone, an effect that was significantly retarded (particularly at earlier time points) in the presence of the Akt inhibitor. Although *Klf6* is not the most commonly discussed FOXO target gene, a recent whole transcriptome analysis of FOXO-deficient liver endothelial cells identified *Klf6* as one of the top two most significantly down-regulated genes with the highest number of conserved FOXO-binding elements ^40^. Moreover, a ChIP-based study identified *Klf6* as a direct transcriptional target of FOXO1 ^41^. Further study will be necessary to determine to what extent the global effects of Akt on CD4 ^+^ T cell biology are due specifically to effects on FOXO vs. NF-κB.

Of note, Myc target genes were also found enriched in the subset of Akt-dependent genes after T cell activation. It is well known that Myc is associated with cell activation. However, it is now thought that Myc is not an on-off specifier of a particular transcriptional program(s) but is a universal amplifier of gene expression, increasing output at all active promoters ^56^. Relevant for our findings, N-Myc was reported to function as a regulator of cell growth by stimulating expression of genes functioning in ribosome biogenesis and protein synthesis ^57^. Akt was also reported to cooperate with c-Myc to increase ribosome biogenesis and cell growth, which includes the synthesis of rRNA and ribosomal proteins, processing of 45S rRNA, and assembly of functional ribosomal subunits ^52^. In our present study, we showed that Akt inhibition decreased the expression of many ribosomal proteins, including components of both the small (40S) and large (60S) ribosomal subunits. Putting together these disparate observations, it may be that in activated T helper cells, one of the mechanisms through which Akt broadly regulates ribosomal subunit transcription is by activating Myc.

Akt is also known to enhance ribosomal protein production through the mammalian target of rapamycin (mTOR) pathway. However, it was reported that direct inhibition of Akt only weakly suppressed the phosphorylation of S6 and S6K1 in CTLs ^10^ and Akti1/2 could only weakly suppress the CD3/CD28 stimulation dependent phosphorylation of S6 in Jurkat T cells and primary helper T cells (unpublished data from our lab). Akt could signal through mTORC1-dependent and independent mechanisms to promote rDNA transcription in mammalian cells ^52^. Dissecting out the mTOR-independent mechanism that Akt utilizes to regulate ribosomal biogenesis is crucial to understand the therapeutic response to Akt inhibitors in cancer.
