# Supplementary material for: Global identification of genes and pathways regulated by Akt during activation of T helper cells
Source: F1000Res. 2013 May 15;2:109. Originally published 2013 Apr 15. [Version 2] doi: 10.12688/f1000research.2-109.v2 (PMC3924950; doi:10.12688/f1000research.2-109.v2)
Supplement: Peer review history

# Peer review: Referee response for version 2

**DOI:** 10.5256/f1000research.1457.r1602

**Published:** 2014-03-04

**Authors:** David Finlay

**Affiliations:** 1 School of Biochemistry and Immunology, Trinity College Dublin, Dublin, Ireland

While some of my concerns that I stated in my first report remain, the authors have clarified most of the issues that I highlighted. This article contributes to our knowledge of Akt in CD4 T cells and so I am happy to approve it.

I have read this submission. I believe that I have an appropriate level of expertise to confirm that it is of an acceptable scientific standard.

# Peer review: Referee response for version 2

**DOI:** 10.5256/f1000research.1457.r3257

**Published:** 2014-01-22

**Authors:** Ursula Bommhardt

**Affiliations:** 1 Otto-von-Guericke University Magdeburg, Magdeburg, Germany

I have read the revised and improved version of the manuscript and approve it.

I have read this submission. I believe that I have an appropriate level of expertise to confirm that it is of an acceptable scientific standard.

# Peer review: Referee response for version 2

**DOI:** 10.5256/f1000research.1457.r3241

**Published:** 2014-01-21

**Authors:** Michael Gold

**Affiliations:** 1 Microbiology & Immunology, University of British Columbia, Vancouver, BC, Canada

I have looked through this revised version and approve of this one as well, with some of the same caveats that are indicated in my initial review. Nevertheless, this is a valuable addition to the field and data that will be of use to researchers, so I approve of its publication in its present form.

I have read this submission. I believe that I have an appropriate level of expertise to confirm that it is of an acceptable scientific standard.

# Peer review: Referee response for version 1

**DOI:** 10.5256/f1000research.1303.r957

**Published:** 2013-05-20

**Authors:** Michael Gold

**Affiliations:** 1 Microbiology & Immunology, University of British Columbia, Vancouver, BC, Canada

This article provides useful information for further assessing the role of Akt signaling in the activation of CD4+ T cells. The microarray experiments that assess the effect of an Akt inhibitor compound on gene regulation by TCR/CD28 co-stimulation are well done and the accompanying bioinformatics analyses are quite sophisticated such that identified changes in gene expression needed to have passed multiple statistical tests.

Although a good starting point for further studies, the data presented have several limitations. First, the experiments were carried out entirely with a T cell line. It would have been straightforward, and of significant value, to confirm some of the key gene expression changes in primary T cells stimulated with anti-CD3/anti-CD28 in the presence or absence of the Akt inhibitor. It is of interest that two of the Akt-dependent genes identified in this report are IL-13 and IL-4, suggesting that Akt is critical for the effector functions of Th2 cells. It would be worthwhile to discuss the role of Akt signaling in Th2 polarization versus Th2 function and to refer to, or propose, further studies to address this question. 

Second, as for any study employing chemical inhibitors, it would have been useful for the authors to briefly review selectivity of this compound as well as known off-target effects on other kinases. In this regard, confirming some of the key gene expression changes by using other commercially available Akt inhibitors with different off-target effects would help confirm the dependence on Akt. 

Similarly, the conclusion that many of the Akt-regulated genes may be co-regulated by NF-kB or Myc could have been tested in a limited fashion.

Despite these limitations, which will likely be dealt with in follow-up studies by these authors and others, this article provides useful data that will stimulate further work and discussion.

I have read this submission. I believe that I have an appropriate level of expertise to confirm that it is of an acceptable scientific standard.

# Peer review: Referee response for version 1

**DOI:** 10.5256/f1000research.1303.r925

**Published:** 2013-05-03

**Authors:** Ursula Bommhardt

**Affiliations:** 1 Otto-von-Guericke University Magdeburg, Magdeburg, Germany

In this publication, Cheng and Kane mapped the gene expression profile of a Th2 cell line stimulated with cross-linked CD3/CD28 Abs in the presence of Akti1/2 inhibitor using microarray and qRT-PCR analysis. They found gene expression that is not moderately or strongly affected by Akti1/2. Akt-dependent genes included cytokines (like IL-4, IL-5 and IL-13), chemokines (CCL1, CCL3 and CCL4), and a number of genes involved in the biosynthesis and function of ribosomes. Major Akt-dependent transcription factors included members of the NFkappaB family, c-myc and their target genes.

The study provides interesting and novel information on Akt-dependent signalling and Akt target genes which will be very useful for the further understanding of Akt function in T cells. In this line, previous publication by Patra *et al*. on myrAkt expression in activated CD4+ T cells had shown increased expression of Th1 as well as Th2 cytokines, including IL-10, IL-4, IL-13 ( Patra *et al*., JI, 2004). Thus, the data of the current study support those previous results. The authors should include this publication in their citation list as well as other publication from the groups of  P. Ohashi, C. Thompson and others with regard to Akt, NFkappaB and tumorigenesis.

I have read this submission. I believe that I have an appropriate level of expertise to confirm that it is of an acceptable scientific standard.

# Peer review: Referee response for version 1

**DOI:** 10.5256/f1000research.1303.r910

**Published:** 2013-04-24

**Authors:** David Finlay

**Affiliations:** 1 School of Biochemistry and Immunology, Trinity College Dublin, Dublin, Ireland

This paper aims to determine to what extent Akt regulates the transcriptional landscape in TCR/CD28 activated CD4 T cells. While a considerable amount is known regarding Akt regulation of gene expression in activated CD8 cytotoxic T cells ( McIntyre *et al.*, 2011 Immunity 34(2) 224-236) less information exists with respect to CD4 T cells. Indeed it is true, as the authors say, that Akt may have distinct roles in different T cell subsets. Therefore, determining the role of Akt in controlling the transcriptome in CD4 T cell subsets addressed an important and relevant question. However, the data would have been of a much higher value had this study been carried out in primary T cells rather than a CD4 T cell line. There is no reason that using primary CD4 T cells would not have been feasible and I struggle to understand why this was not done. Therefore, this data needs to be viewed with this major caveat in mind.

I have substantial concerns regarding the concentration of Akti1/2 that is used in this study. In CD8 T cells 1µM Akti1/2 is sufficient to completely block all Akt activity. This study uses 10µM, 10 times the dose used in CD8 T cell. When using kinase inhibitors, it is extremely important to use the lowest dose that effectively inhibits the kinase activity, as excessive doses can have increased off target effects. For example it has been shown that Akti1/2 has multiple off target effects at 10µM when screened against a panel of 80 kinases ( Bain J, *et al.,* 2007, Biochem. J. 408, 297-315). Additionally, in CD8 Cytotoxic T cells, 10µM Akti1/2 is toxic and compromises cell viability, a clear demonstration of off target effects (unpublished data). The authors should demonstrate that they are using the minimum effective dose and consider what aspect of the results that they present in this paper might be due to off target effects of the Akti1/2 compound.

With respect to their data, the authors discuss the role of Foxo transcription factors as Akt substrates and there role in CD4 T cells despite the fact that they have little to no evidence that the activity of Foxo transcription factors are altered in response to Akt inhibition in this model. They state Klf6 as a Foxo target. **The authors must clearly reference the work that describes Klf6 as a Foxo Target**. At best Klf6 is an obscure Foxo target gene. There are numerous well defined Foxo targets in T cells, Klf2 for example, yet the absence of these Foxo target genes in the Akti1/2 regulated gene lists is not discussed at all.

A minor point, in the text it is stated that CREB was one of the transcription factors predicted by TFactS to be altered in activity (bottom of page 5), yet CREB does not appear in the corresponding figure (Figure 6).

I have read this submission. I believe that I have an appropriate level of expertise to confirm that it is of an acceptable scientific standard, however I have significant reservations, as outlined above.
